# Does stomatal patterning in amphistomatous leaves minimize the CO_2_ diffusion path length within leaves?

**DOI:** 10.1093/aobpla/plae015

**Published:** 2024-03-20

**Authors:** Jacob L Watts, Graham J Dow, Thomas N Buckley, Christopher D Muir

**Affiliations:** School of Life Sciences, University of Hawai’i at Mānoa, Honolulu, HI 96822, USA; Ecology and Evolutionary Biology, University of Colorado, Boulder, CO 80309, USA; Department of Crop Science and Production Systems, NIAB, Cambridge CB3 0LE, UK; Department of Plant Sciences, University of California, Davis, CA 95616, USA; School of Life Sciences, University of Hawai’i at Mānoa, Honolulu, HI 96822, USA; Department of Botany, University of Wisconsin, Madison, WI 53706, USA

**Keywords:** Amphistomy, *Arabidopsis thaliana*, CO_2_ diffusion, finite element method, optimality, photosynthesis, stomata

## Abstract

Photosynthesis is co-limited by multiple factors depending on the plant and its environment. These include biochemical rate limitations, internal and external water potentials, temperature, irradiance and carbon dioxide ( ${CO}_{\text{2}}$). Amphistomatous leaves have stomata on both abaxial and adaxial leaf surfaces. This feature is considered an adaptation to alleviate ${CO}_{\text{2}}$ diffusion limitations in productive environments as the diffusion path length from stomate to chloroplast is effectively halved in amphistomatous leaves. Plants may also reduce ${CO}_{\text{2}}$ limitations through other aspects of optimal stomatal anatomy: stomatal density, distribution, patterning and size. Some studies have demonstrated that stomata are overdispersed compared to a random distribution on a single leaf surface; however, despite their prevalence in nature and near ubiquity among crop species, much less is known about stomatal anatomy in amphistomatous leaves, especially the coordination between leaf surfaces. Here, we use novel spatial statistics based on simulations and photosynthesis modelling to test hypotheses about how amphistomatous plants may optimize ${CO}_{\text{2}}$ diffusion in the model angiosperm Arabidopsis thaliana grown in different light environments. We find that (i) stomata are overdispersed, but not ideally dispersed, on both leaf surfaces across all light treatments; (ii) the patterning of stomata on abaxial and adaxial leaf surfaces is independent and (iii) the theoretical improvements to photosynthesis from abaxial–adaxial stomatal coordination are miniscule (≪1%) across the range of feasible parameter space. However, we also find that (iv) stomatal size is correlated with the mesophyll volume that it supplies with ${CO}_{\text{2}}$, suggesting that plants may optimize ${CO}_{\text{2}}$ diffusion limitations through alternative pathways other than ideal, uniform stomatal spacing. We discuss the developmental, physical and evolutionary constraints that may prohibit plants from reaching this theoretical adaptive peak of uniform stomatal spacing and inter-surface stomatal coordination. These findings contribute to our understanding of variation in the anatomy of amphistomatous leaves.

## Introduction

Stomatal anatomy (e.g. size, density, distribution and patterning) and movement regulate gas exchange during photosynthesis, namely ${CO}_{2}$ assimilation and water loss through transpiration. Since waxy cuticles are mostly impermeable to ${CO}_{2}$ and $H_{2}$O, stomata are the primary entry and exit points through which gas exchange occurs despite making up a small percentage of the leaf area (Lange *et al.* 1971). Stomata consist of two guard cells that open and close upon changes in turgor pressure or hormonal cues (McAdam and Brodribb 2016). The stomatal pore leads to an internal space known as the substomatal cavity where gases contact the mesophyll. Once in the mesophyll, ${CO}_{2}$ diffuses throughout a network of intercellular air space (IAS) and into mesophyll cells where ${CO}_{2}$ assimilation (A) occurs within the chloroplasts (Lee and Gates 1964). Stomatal conductance and transpiration are determined by numerous environmental and anatomical parameters such as vapor pressure deficit (VPD), irradiance, temperature, wind speed, leaf water potential, IAS geometry, mesophyll cell anatomy and stomatal anatomy. The latter of these is the focus of this study, with the discussion of other interacting variables.

Many successful predictions about stomata and other $C_{3}$ leaf traits can be made by hypothesizing that natural selection should optimize ${CO}_{2}$ gain per unit of water loss for any given set of environmental parameters, including their variability (Cowan and Farquhar 1977; Buckley *et al.* 2017; Sperry *et al.* 2017). Total stomatal area (size × density) is optimized for operational conductance ($g_{\text{s,op}}$) rather than maximum conductance ($g_{\text{s,max}}$) such that stomatal apertures are most responsive to changes in the environment at their operational aperture (Franks *et al.* 2012; Liu *et al.* 2021). Stomatal aperture can compensate for suboptimal stomatal densities to an extent (Büssis *et al.* 2006), but stomatal density and size ultimately determine a leaf’s theoretical $g_{\text{s,max}}$ (Sack and Buckley 2016), which is proportional to $g_{\text{s,op}}$ under typical conditions (McElwain *et al.* 2016; Murray *et al.* 2020). In addition, low stomatal densities lead to irregular and insufficient ${CO}_{2}$ supply and reduced photosynthetic efficiency in leaf areas far from stomata (Morison *et al.* 2005; Pieruschka *et al.* 2006), while high stomatal densities can reduce water use efficiency (WUE) (Büssis *et al.* 2006) and incur excessive metabolic costs (de Boer *et al.* 2016; Deans *et al.* 2020). Stomatal density positively co-varies with irradiance during leaf development and negatively co-varies with ${CO}_{2}$ concentration (Gay and Hurd 1975; Schoch et al. 1980; Woodward 1987; Royer 2001), consistent with optimality predictions. In most species, stomata occur only on the abaxial (usually lower) leaf surface; but amphistomy, the occurrence of stomata on both abaxial and adaxial leaf surfaces, is also prevalent in high-light environments with constant or intermittent access to sufficient water (Mott *et al.* 1982; Jordan *et al.* 2014; Muir 2018; Drake *et al.* 2019; Muir 2019). Amphistomy effectively halves the ${CO}_{2}$ diffusion path length and boundary layer resistance by doubling boundary layer conductance (Parkhurst 1978; Mott and Michaelson 1991; Harrison *et al.* 2020). Ab- and adaxial leaf surfaces were found to function independent of one another in wheat, an important crop, with the adaxial surface demonstrating higher photosynthetic capacity (Wall *et al.* 2022). These results highlight the utmost importance of amphistomy for some plants.

Despite the success of optimality predictions, stomatal anatomy may be partially constrained by physical and developmental limits on phenotypic expression (Croxdale 2000; Harrison *et al.* 2020; Muir *et al.* 2023). A number of physical and developmental processes constrain stomatal anatomy trait space. For example, almost all stomata follow the one-cell spacing rule to maintain proper stomatal functioning as guard cell movement requires the rapid exchange of ions with neighboring epidermal cells (i.e. subsidiary cells) (Geisler et al. 2000; Dow *et al.* 2014). This would prevent stomata from being strongly clustered; however, some species (notably in *Begonia*) appear to benefit from the overlapping vapor shells caused by stomatal clustering in dry environments (Yi Gan et al. 2010; Lehmann and Or 2015; Papanatsiou *et al.* 2017). Historically, stomatal patterning in eudicot angiosperms was thought to be random with an exclusionary distance surrounding each stomate (Sachs 1974); however, the developmental controls of stomatal patterning are more complex. Croxdale (2000) reviews three developmental theories that attempt to explain stomatal patterning in angiosperms: inhibition, cell lineage and cell cycle, ultimately arguing for a cell cycle-based control of stomatal patterning. Pillitteri and Torii (2012) review the short- and long-distance signalling pathways associated with stomatal spacing and development, which include cell to cell communication and whole-plant integration to ensure the proper spacing of stomata across a single leaf surface depending on environmental ques. Much less is known about the development of stomata on the adaxial leaf surface in amphistomatous plants. Stomatal size is additionally constrained by genome size with larger genomes leading to larger minimum guard cell size (Jordan *et al.* 2015; Roddy *et al.* 2020). Despite these limitations, ecophysiological theory still predicts optimal stomatal anatomy, the details of which are discussed below.

The patterning and spacing of stomata on the leaf affect photosynthesis in $C_{3}$ leaves by altering the ${CO}_{2}$ diffusion path length from stomata to sites of carboxylation in the mesophyll. Maximum photosynthetic rate ($A_{\text{max}}$) in $C_{3}$ plants is generally co-limited by biochemistry and diffusion, but modulated by light availability (Parkhurst and Mott 1990; Manter 2004; Carriquí *et al.* 2015). Low light decreases ${CO}_{2}$ demand by limiting electron transport rate, leading to relatively high internal ${CO}_{2}$ concentration ($C_{\text{i}}$) and low $A_{\text{max}}$ (Kaiser *et al.* 2016). In contrast, well-hydrated leaves with open stomata in high light, photosynthesis is often limited by ${CO}_{2}$ supply as resistances from the boundary layer, stomatal pore, sub-stomatal cavity and mesophyll can result in insufficient CO$C_{2}$ supply at the chloroplast to maximize photosynthesis (Farquhar *et al*. 1980; Lehmeier *et al.* 2017). In this study, we focus primarily on how stomatal patterning affects diffusion.

Assuming uniform mesophyll diffusion resistance in all directions (homogenous porous medium), an ideal stomatal anatomy can be predicted. To maximize ${CO}_{2}$ supply from the stomatal pore to chloroplasts, stomata should be uniformly distributed in an equilateral triangular grid on the leaf surface so as to minimize stomatal number and ${CO}_{2}$ diffusion path length (Parkhurst 1994). An equilateral triangular grid is ideal because it maximizes the average distance between stomata, for a given stomatal density and thereby minimizes the average distance between any point in the mesophyll to its nearest stomate. Assuming a homogenous mesophyll, this is the most efficient pattern to supply ${CO}_{2}$ to a leaf volume.

Such an assumption, though an oversimplification, is a powerful tool for photosynthesis modelling, and may provide insight into how real leaves diverge from this. In real leaves, as the diffusion rate of ${CO}_{2}$ though liquid is approximately $10^{4}\times$ slower than ${CO}_{2}$ diffusion through air, mesophyll resistance is generally thought to be primarily limited by liquid diffusion (Aalto and Juurola 2002; Evans *et al.* 2009), but diffusion through the IAS has also been shown to be a rate-limiting process because the tortuous, disjunct nature of the IAS can greatly increase diffusion path lengths (Harwood *et al.* 2021). In addition, tortuosity is higher in horizontal directions (parallel to leaf surface) than vertical directions (perpendicular to leaf surface) because of the cylindrical shape and vertical arrangement of palisade mesophyll cells (Earles *et al.* 2018; Harwood *et al.* 2021). However, the ratio of lateral to vertical diffusion rate is still largely unknown and may be a highly variable trait in leaves (Morison *et al.* 2005; Pieruschka 2005; Pieruschka *et al.* 2006; Morison and Lawson 2007). Depending on the thickness of the leaf, porosity of the leaf mesophyll, tortuosity of the IAS and lateral to vertical diffusion rate ratio, minimizing diffusion path length for ${CO}_{2}$ via optimally distributed stomata may yield significant increases in ${CO}_{2}$ supply for photosynthesis and higher $A_{\text{max}}$. Or plants may simply coordinate the development of stomata and mesophyll IAS to reach another optimal solution that does not rely on uniformly distributed stomata (Baillie and Fleming 2020).

**Figure 1:**
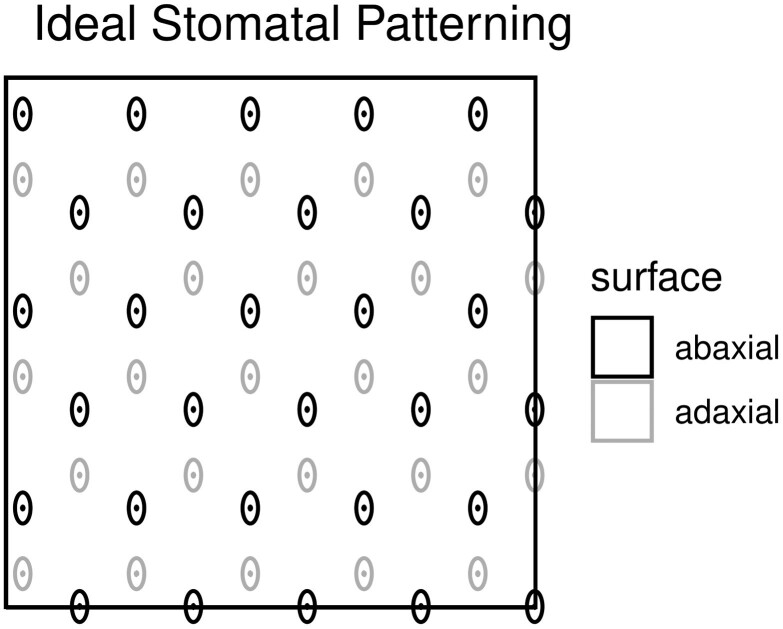
Idealized amphistomatous stomatal grid with uniform stomatal patterning and perfect abaxial–adaxial coordination.

We hypothesized that in the absence of any constraint and assuming homogenous mesophyll diffusion resistance, natural selection will favor stomatal patterning and distribution to minimize the diffusion path length. In amphistomatous leaves, this would be accomplished by (i) a uniform, equilateral triangular distribution of stomata on both abaxial and adaxial leaf surfaces and (ii) coordinated stomatal spacing on each surface that offsets the position of stomata (Fig. 1). Coordination between leaf surfaces is defined, in this study, as the occurrence of stomata in areas farthest from stomata on the opposite leaf surface. Additionally, because ${CO}_{2}$ is more limiting for photosynthesis under high light, we hypothesize that in high light (iii) there should be more stomata and (iv) stomata should be more overdispersed (closer to equilateral triangular grid) compared to a random distribution than in low light. Finally, since, in measures of whole leaves, stomatal area rather than stomatal density is optimized for operational conductance, we hypothesize that (v) stomatal length (and hence its area) will be positively correlated with the area of the leaf surface to which it is spatial closest as defined by Voronoi tessellation techniques. We refer to this as the ‘stomatal zone’, the leaf area surrounding a focal stomate closest to that stomate and, therefore, the zone it supplies with ${CO}_{2}$). This way, each stomate may be optimally sized relative to the mesophyll volume it supplies. Hypothesis 3 is already well supported in many species (Poorter *et al.* 2019), but it is useful here to confirm that light treatments induced plasticity in the expected direction.

To test these hypotheses, we grew the model plant *Arabidopsis thaliana* in high, medium and low light and measured stomatal density, size and patterning on both leaf surfaces and spatial coordination between them. We use Voronoi tessellation techniques to calculate stomatal zones. We also used a 2-D porous medium approximation of ${CO}_{2}$ diffusion and photosynthesis to predict the photosynthetic advantage of optimal versus suboptimal coordination in stomatal coordination between surfaces. Specifically, we predicted that traits that affect diffusion path length (leaf thickness, stomatal density, leaf porosity), diffusion rate (determined by temperature, pressure) and ${CO}_{2}$ demand (Rubisco concentration, light) would modulate the advantage of optimal stomatal arrangement following the relationships outlined in Table 1. Here, we integrate over reasonable parameter space to determine the ecophysiological context most likely to favor stomatal coordination in amphistomatous leaves.

**Table 1:** A summary of the hypothesized relationships between leaf traits and environmental conditions and photosynthetic advantage of stomatal spatial coordination in amphistomatous leaves. We also list the associated symbol and parameter range of model variables tested for their effect on coordination advantage (Equation (4)) using a 2-D porous medium approximation. We used regularly spaced values within each range and simulated across all combinations. Here, we converted model units to more conventional units (e.g. m to μm). $I_{0}$: PPFD incident on the leaf surface; $\varphi_{\text{pal}}$: fraction of intercellular airspace (aka porosity), palisade; $T_{\text{leaf}}$: leaf thickness; U: interstomatal distance.

Trait	Relationship	Symbol	Parameter range	Units
Leaf thickness	+	$T_{\mathrm{leaf}}$	101–501	μ m
Interstomatal				
distance	+	U	17–169	μ m
Leaf porosity	–	$\varphi_{\mathrm{pal}}$	0.1–0.3	$m^{3}$ airspace $m^{-3}$
				Leaf
Light	+	$I_{0}$	50–1000	μ mol $m^{-2}$ $s^{-1}$

## Materials and Methods

### Data preparation

Plant material, growth conditions and three-dimensional confocal imaging are described in Dow *et al.* (2017). Briefly, Columbia (Col-0) ecotype of *A. thaliana* plants were grown in three different light environments: low light (PAR = 50 $\mu \,\text{mol}\,{\text{m}}^{-2}\,{\text{s}}^{-1}$), medium light (100 $\mu \,\text{mol}\,{\text{m}}^{-2}\,{\text{s}}^{-1}$) and high light (200 $\mu \,\text{mol}\,{\text{m}}^{-2}\,{\text{s}}^{-1}$). PAR stands for photosynthetically active radiation. *A. thaliana* responds strongly to light levels over this range (Bailey *et al.* 2001), though natural populations in open canopies can experience $\text{PAR}>800\,\mu \,\text{mol}\,{\text{m}}^{-2}\,{\text{s}}^{-1}$ (Callahan and Pigliucci 2002). Seeds were surface-sterilized and stratified at 4 *C for 3–5 d in 0.15 % agarose solution and then sown directly into Pro-Mix HP soil (Premier Horticulture; Quakerstown, PA, USA) and supplemented with Scott’s Osmocote Classic 14-14-14 fertilizer (Scotts-Sierra, Marysville, OH, USA). At 10–14 d, seedlings were thinned so only one seedling per container remained. Plants were grown to maturity in growth chambers where the conditions were as follows: 16: 8 h, 22: 20 *C, day:night cycle. Imaging of the epidermis and internal leaf structures was performed using a Leica SP5 confocal microscope (Leica Microsystems, Wetzlar, Germany) with the protocol developed by Wuyts *et al.* (2010) with the additional modification described in Dow *et al.* (2017). We captured 132 images in total, making 66 abaxial–adaxial image pairs. Images were square with an area of 0.386 ${\mathrm{mm}}^{2}$. We measured stomatal position and length using ImageJ (Schneider *et al.* 2012). A number of synthetic leaf surface data sets were also simulated (details below) to generate null distributions against which to test our hypotheses and to avoid any methodological influence on our results (e.g. boundary effects when calculating stomatal patterning). All synthetic leaf surfaces were simulated based on the size of the real leaf images and stomatal densities matched those of real leaf images.

### Single surface analyses

We compared observed stomatal patterning to an ideal pattern (uniform equilateral triangular grid) and a null model (random uniform distribution). The terminology is unfortunately confusing because the word ‘uniform’ is used in different ways. A uniform equilateral triangular grid means that the distance between stomata is uniform; a random uniform distribution means that a stomate has an equal probability (i.e. uniform) of occurring anywhere on the leaf surface. To limit confusion, we refer to the ideal pattern (equilateral triangle grid) as uniform and the null pattern (random uniform) as random. When observed stomatal patterns are more dispersed than expected under random patterning, we refer to this as overdispersed. Note, however, that overdispersed compared to random is still less dispersed than ideal because the ideal pattern is maximally dispersed.

We tested whether stomata overdispersed by comparing each observed, real leaf stomatal pattern to an array of synthetic data simulated from a random distribution. For each observed leaf surface image with n stomata, we generated $10^{3}$ synthetic surfaces with n stomata uniformly randomly distributed on the surface. For each sample image, we compared the observed Nearest Neighbor Index (NNI) to the null distribution of NNI values calculated from the synthetic data set. NNI is the ratio of observed mean distance (${\bar{D}}_{O}$) to the expected mean distance (${\bar{D}}_{E}$) where ${\bar{D}}_{E}$ is:


$${\bar{D}}_{E}=\frac{0.5}{\sqrt{A_{\text{leaf}}/n_{\text{stomata}}}}.$$ (1)

$A_{\text{leaf}}$ is leaf area visible in the sampled field and $n_{\text{stomata}}$ is the number of stomata. ${\bar{D}}_{E}$ is the theoretical average distance to the nearest neighbour of each stomate if stomata were uniformly randomly distributed (Clark and Evans 1954). ${\bar{D}}_{O}$ calculated for each synthetic data set is:


$${\bar{D}}_{O}=\frac{\sum_{i=1}^{n_{\text{stomata}}}d_{i}}{n_{\text{stomata}}},$$ (2)

where $d_{i}$ is the distance between ${\text{stomate}}_{i}$ and its nearest neighbour. We calculated NNI using the *R* package **spatialEco** (version 2.0.2) (Evans and Murphy 2023). The observed stomatal distribution is overdispersed relative to a random distribution if the observed NNI is greater than 95 % of the synthetic NNI values (one-tailed test). We adjusted P-values to account for multiple comparisons using the Benjamini–Hochberg (Benjamini and Hochberg 1995) false discovery rate procedure implemented in the *R* package **multtest** (version 2.56.0) (Pollard *et al.* 2005).

For each sample image, we also simulated $10^{3}$ synthetic leaf surfaces with n stomata ideally, uniformly dispersed in an equilateral triangular grid. To account for uncertainty in the stomatal density of each sample image with n stomata, we integrated over plausible stomatal densities and then conditioned on synthetic leaf surfaces with exactly n stomata. The simulated stomatal count was drawn from a Poisson distribution with the mean parameter λ drawn from a Gamma distribution with shape n and scale 1 (λ∼Γ⁢(n,1)). Γ⁢(n,1) is the posterior distribution of λ with a flat prior distribution. This integration was necessary to remove any artefacts of uncertainty in the true stomatal density of the sample leaves.

We developed a dispersion index DI to quantify how close observed stomatal patterning is to random versus ideally patterned in an equilateral triangular grid. DI varies from zero to one, where zero is random and one is ideally patterned:


$$\mathrm{DI}=\frac{\mathrm{NNI}-\text{median}\,\left({\mathrm{NNI}}_{\mathrm{random}}\right)}{\text{median}\,\left({\mathrm{NNI}}_{\mathrm{ideal}}\right)-\text{median}\,\left({\mathrm{NNI}}_{\mathrm{random}}\right)}.$$ (3)

NNI is calculated for each sample image as described above; $\text{median}\,\left({\mathrm{NNI}}_{\mathrm{random}}\right)$ and $\text{median}\,\left({\mathrm{NNI}}_{\mathrm{uniform}}\right)$ are calculated from the synthetic data specific to each sample image as described above. We tested whether light treatment affects DI and stomatal density ($D_{S}$) using analysis of variance (ANOVA).

**Figure 2:**
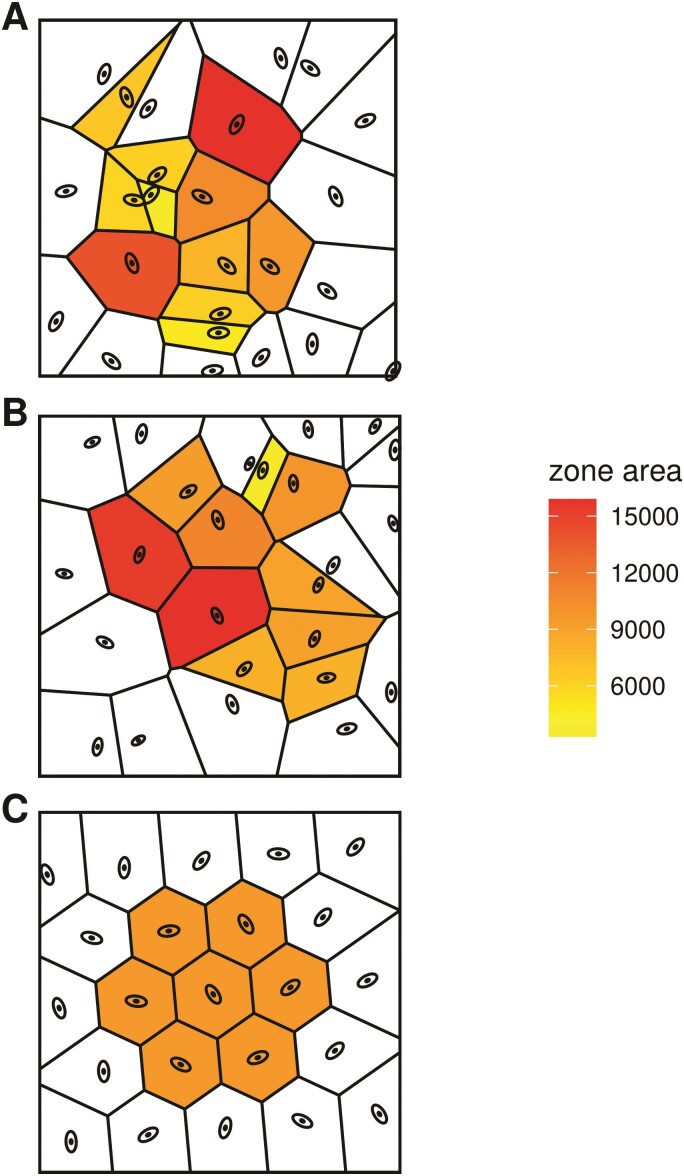
Examples of synthetic and real leaf surfaces. (A) Uniform random synthetic leaf surface; (B) example of real leaf surface; (C) uniformly distributed synthetic leaf surface. The zone defined by each stomate was calculated with voronoi tessellation and correlated with stomatal length in real leaves.

Finally, we examined the relationship between stomatal zone area and stomatal length using a Bayesian linear mixed-effects model fit with the *R* package **brms** (version 2.20.4) (Bürkner 2017, 2018) and *Stan* version (2.33.1) (Stan Development Team 2023). Stomatal zone area was calculated using Voronoi tessellation (e.g. Fig. 2). The stomatal zone area, $S_{\text{area}}$, is the region of the leaf surface whose distance to stomate, S, is less than the distance to any other stomate, S. Stomatal length was measured in ImageJ (Schneider *et al.* 2012). We modelled fixed effects of surface, light treatment, stomatal length and their 2- and 3-way interactions on $\sqrt{S_{\text{area}}}$. We included random intercepts, random effects of surface, random slopes and random surface-by-slope interactions within both plant and individual to account for nonindependence of stomata within the same plant or individual. We also modelled residual variance as a function of light treatment. We sampled the posterior distribution from 4 chains with 1000 iterations each after 1000 warmup iterations. We calculated convergence diagnostics ($\hat{R}$) and effective sample sizes following Vehtari *et al.* (2021). We estimated the marginal slope and 95 % highest posterior density (HPD) intervals between stomatal length and $\sqrt{S_{\text{area}}}$ using the *emtrends* function in the *R* package **emmeans** (version 1.10.0) (Lenth 2023).

### Paired abaxial and adaxial surface analysis

To test whether the position of ab- and adaxial stomata are coordinated, we compared the observed distribution to a null distribution where the positions on each surface are random. For each pair of surfaces (observed or synthetic), we calculated the distance squared between each pixel of the surface to the nearest stomatal centroid with the *R* package **raster** (version 3.6.26) (Hijmans 2023). We refer to this as the ‘nearest stomatal distance’ or NSD. Then we calculated the pixel-wise Pearson correlation coefficient. If stomatal positions on each surface are coordinated to minimize the distance between mesophyll and the nearest stomate, then we expect a negative correlation. A pixel that is far from a stomate on one surface should be near a stomate on the other surface (Fig. 1). We generated a null distribution of the correlation coefficient by simulating $10^{3}$ synthetic data sets for each observed pair. For each synthetic data set, we simulated stomatal position using a random uniform distribution, as described above, matching the number of stomata on abaxial and adaxial leaf surfaces to the observed data. Stomatal positions on each surface are coordinated if the correlation coefficient of the NSD between observed ab- and adaxial surfaces is greater than 95 % of the synthetic correlation values (one-tailed test).

### Modeling photosynthesis

We modelled photosynthesis ${CO}_{2}$ assimilation rate using a spatially explicit two-dimensional reaction-diffusion model using a porous medium approximation (Parkhurst 1994) using the finite element method (FEM) following Earles *et al.* (2017). Consider a two-dimensional leaf where stomata occur on each surface in a regular sequence with interstomatal distance U. The main outcome we assessed is the advantage of offsetting the position of stomata on each surface compared to having stomata on the same x position on each surface. With these assumptions, by symmetry, we only need to model two stomata, one abaxial and one adaxial, from x=0 to x=U/2 and from the adaxial surface at y=0 to the abaxial surface at y=L, the leaf thickness. We arbitrarily set the adaxial stomate at x=0 and toggled the abaxial stomata position between x=U/2 (offset) or x=0 (below adaxial stomate). The ‘coordination advantage’ of offset stomatal position on each surface is the photosynthetic rate of the leaf with offset stomata compared to that with stomata aligned in the same x position:


$$\text{coordination advantage}=\frac{A_{\text{offset}}}{A_{\text{aligned}}}.$$ (4)

We modelled the coordination advantage over a range of leaf thicknesses, stomatal densities, photosynthetic capacities and light environments to understand when offsetting stomatal position on each surface might deliver a significant photosynthetic advantage (Table 1). The complete model description is available in the Supporting Information.

## Results

**Figure 3:**
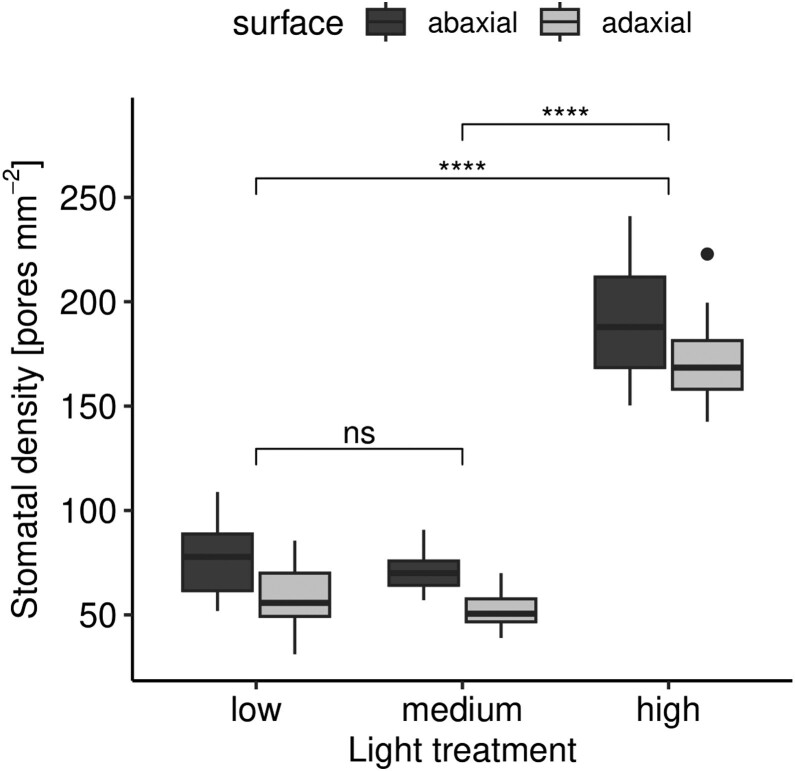
Stomatal density is higher in *A. thaliana* plants grown under high light conditions. We determined the statistical significance between light treatments using Tukey post-hoc tests. * 0.05 > *P* ≥ 0.01; ** 0.01 > *P* ≥ 0.001; *** 0.0001 > *P* ≥ 0.0001; *** *P* <0.0001.

Stomatal density of *A. thaliana* varies among light treatments (ANOVA, $F_{2,126}=682,P=2.58\times 10^{-68}$) because the density is much greater in the high-light treatment (Fig. 3). Density is consistently greater on abaxial leaf surfaces across all light treatments (ANOVA, $F_{1,126}=44.2,P=8.21\times 10^{-10}$; Fig. 3). There is no evidence for an interaction between light treatment and surface (ANOVA, $F_{2,126}=2.75\times 10^{-2},P=0.973$). Leaves are amphistomatous with a mean stomatal density ratio of 0.44.

### Stomatal patterning is non-random, but far from uniform

**Figure 4:**
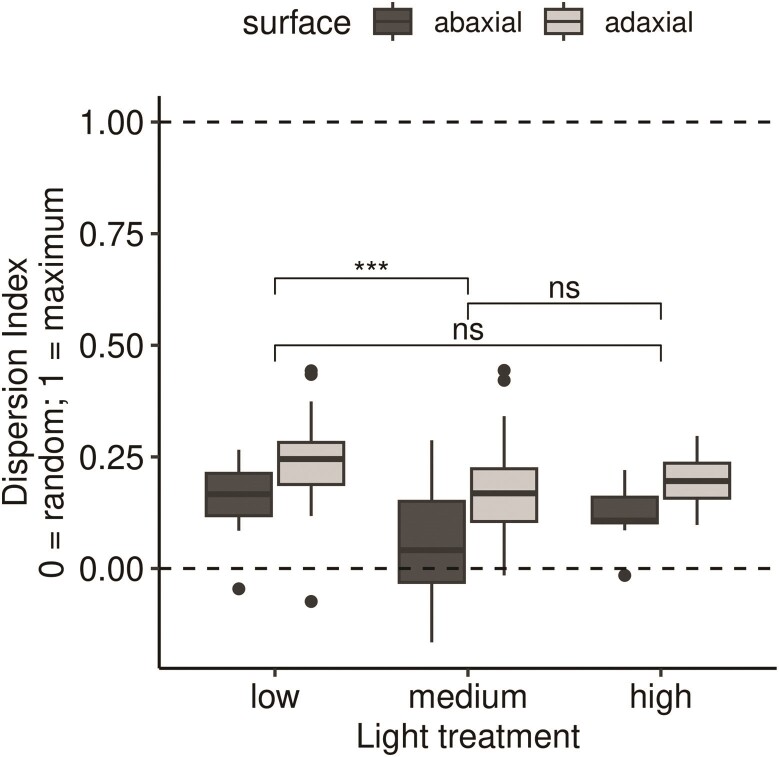
Stomata are more dispersed than expected under the null model of random patterning (dispersion index = 0) but far from a distribution that maximizes the distance between stomata (dispersion index = 1; uniform patterning). We determined statistical significance between light treatments using Tukey post-hoc tests. * 0.05 > *P* ≥ 0.01; ** 0.01 > *P* ≥ 0.001; *** 0.0001 > *P* ≥ 0.0001; ***  *P* <0.0001.

Many leaf surfaces (34 of 132, 25.8 %) are significantly overdispersed compared to a random uniform distribution, but none were close to an ideal, uniform equilateral triangular pattern (dispersion index = 1; Fig. 4). Before controlling for multiple comparisons, 40.9 % are significantly overdispersed. The dispersion index differs significantly among light treatments (ANOVA, $F_{2,126}=7.87,P=6.02\times 10^{-4}$) because the medium light treatment is significantly less than the low treatment (Fig. 4). Dispersion index is consistently greater on adaxial leaf surfaces across all light treatments (ANOVA, $F_{1,126}=29.2,P=3.19\times 10^{-7}$; Fig. 4). There is no evidence for an interaction between light treatment and surface (ANOVA, $F_{2,126}=0.594,P=0.554$).

### No evidence for coordinated stomatal position between surfaces

**Figure 5:**
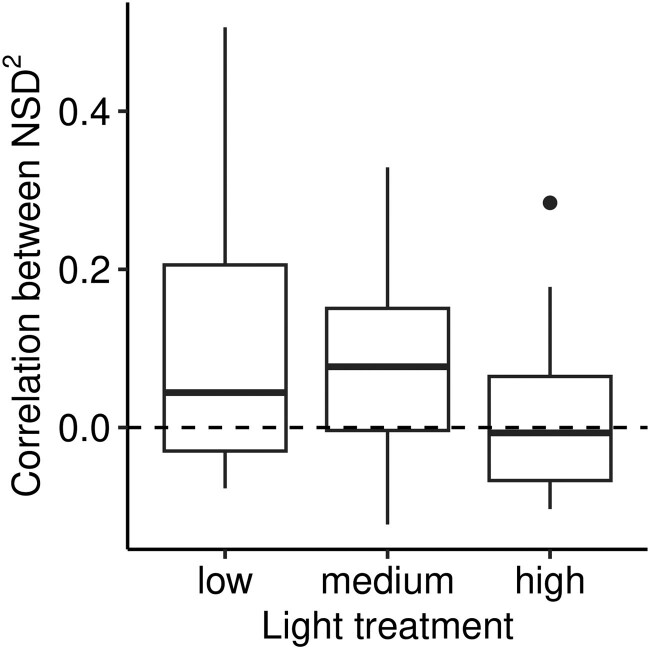
Pixel-wise correlation between NSD squared on paired abaxial and adaxial leaf surfaces. Dashed line indicates zero correlation. Weak positive correlations are not significantly different from zero after correcting for multiple comparisons. The correlation does not differ among light treatments.

There is no evidence of spatial coordination between abaxial and adaxial leaf surfaces. The pixel-wise correlation between the nearest stomatal distance (NSD) squared on paired abaxial and adaxial leaf surfaces is not significantly less than 0 in any of the 66 leaves (Fig. 5). Before controlling for multiple comparisons, 3 % are significantly *positively* correlated. The NSD correlation is not different among light treatments (ANOVA, $F_{2,63}=2.28,P=0.111$; Fig. 5).

### Larger stomata supply larger mesophyll volumes

**Figure 6:**
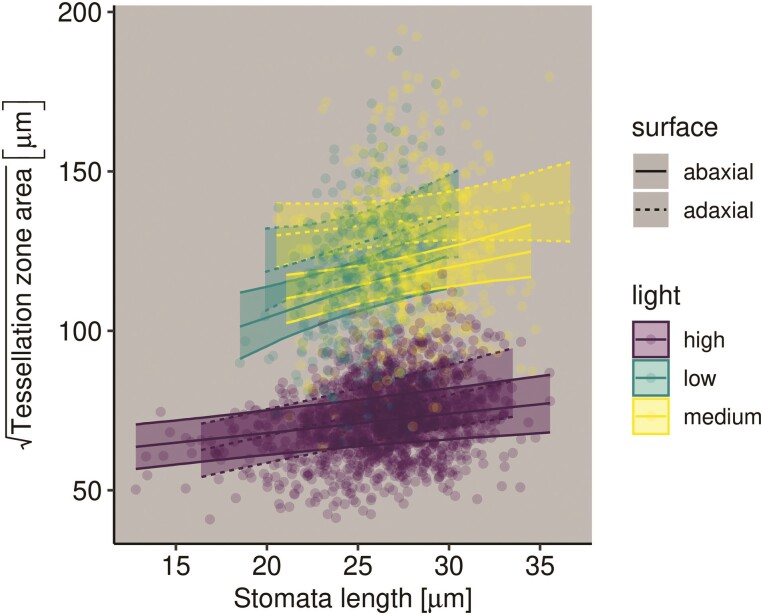
Stomatal length and stomatal zone area are positively correlated. Linear regression lines and 95 % confidence ribbons are from a Bayesian linear mixed-effects model.

All parameters in the Bayesian linear mixed-effects model converged ($\hat{R}<1.01$) and effective sample sizes exceeded $10^{3}$. Across all light treatments and leaf surfaces, stomatal length and stomatal area are weakly positively correlated (Fig. 6). The slope was significantly greater than zero for all abaxial surfaces, but not for the adaxial surface in low and medium light treatments. The estimated marginal slopes and 95 % HPD intervals for each combination of light and surface is: low light, abaxial surface: 1.928 [0.779 to 3.133]; low light, adaxial surface: 1.745 [−0.041 to 3.373]; medium light, abaxial surface: 1.085 [0.328 to 1.957]; medium light, adaxial surface: 0.656 [−0.399 to 1.691]; high light, abaxial surface: 0.597 [0.316 to 0.911]; high light, adaxial surface: 1.269 [0.831 to 1.721].

### Little benefit of coordinated stomatal arrangement

**Figure 7:**
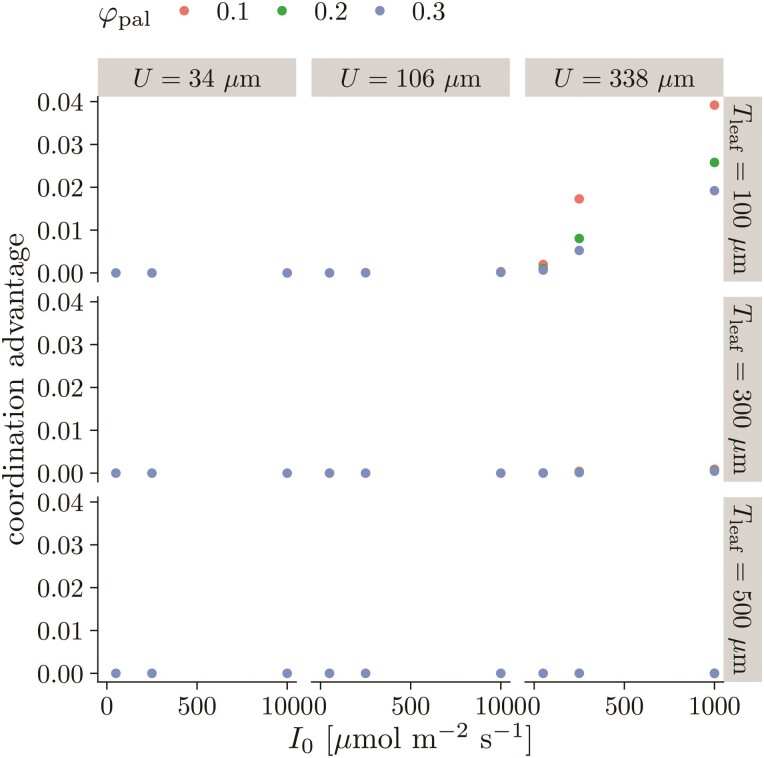
There is little photosynthetic benefit of offsetting stomatal position of each surface based on a 2-D model of photosynthesis. The coordination advantage (Equation (4)) is close to zero under nearly all of the parameter space (Table 1), meaning that the photosynthetic rate of amphistomatous leaves with stomata optimally offset is nearly equal to leaves with stomata on each surface in the same position along the leaf plane. $I_{\text{0}}$: PPFD incident on the leaf surface; $\varphi_{\text{pal}}$: fraction of intercellular airspace (aka porosity), palisade; $T_{\text{leaf}}$: leaf thickness; U: interstomatal distance.

We used the FEM to model ${CO}_{2}$ diffusion within the leaf and photosynthesis as a 2-D porous medium. Across all realistic parts of parameter space, the coordination advantage is much less than 0.01 (Fig. 7). For reference, a log-response of ratio is 0.01 is approximately 1 %. The only exception was for thin leaves ($T_{\text{leaf}}=100\,\mu \,\text{m}$) with few stomata (U=338⁢μ⁢m, which corresponds to a stomatal density of $\approx 10\,{\text{mm}}^{-2}$), where lateral diffusion is major constraint on ${CO}_{2}$ supply. However, such thin leaves with so few stomata are uncommon among $C_{3}$ plants (some CAM plants have low stomatal density (Males and Griffiths 2017)). In other areas of parameter space, lateral diffusion limitations were small relative to those along the ab–adaxial axis [**see Supporting Information—Fig. S1** for a representative model solution].

## Discussion

Stomata cost resources to maintain (Deans *et al.* 2020) and expose leaves to risks such as hydraulic failure (Wang *et al.* 2020) or infection by plant pathogens (Melotto *et al.* 2017). Therefore, leaves should develop enough stomata to adequately supply ${CO}_{2}$ to chloroplasts, but not overinvest. A widespread hypothesis in plant ecophysiology is that natural selection optimizes traits like stomatal size, density and distribution to maximize carbon gain relative to any costs in a given environmental context. In principle, spacing stomata to minimize the average distance between stomatal pores and chloroplasts within the mesophyll should increase carbon gain, all else being equal. However, reducing this distance to its absolute minimum may be constrained by developmental processes or the photosynthetic benefit may be too small to be ‘seen’ by natural selection (i.e. the selection coefficient is less than drift barrier *sensu*  Sung *et al.* (2012)). We also consider that our definition of optimal may be incorrect because it is based on overly simplistic assumptions about leaf mesophyll structure.

We tested five related hypotheses about stomatal spacing in amphistomatous leaves using the model angiosperm *A. thaliana* grown under different light intensities. First, we predicted that stomata on each surface are overdispersed relative to a random distribution, which should increase ${CO}_{2}$ supply. Stomata on each surface are overdispersed (Fig. 4), but are not ideally, uniformly patterned in an equilateral triangular grid as would be optimal to minimize ${CO}_{2}$ diffusion path length and equalize the area supplied by each stomate (Fig. 2). Second, we predicted that an optimal amphistomatous leaf has offset stomata such that stomata are more likely to appear on one leaf surface if there is not a stomata directly opposite it on the other surface as shown in Fig. 1. However, there is no evidence for coordination and the positions on each surface appear independent, regardless of light treatment (Fig. 5). Third, we predicted that plants respond plastically to higher light intensity by increasing stomatal density. *Arabidopsis* plants grown under high light had higher stomatal density than the same genotype grown under low and medium light intensity (Fig. 3). However, we found no support for our fourth prediction that stomatal patterning would be overdispersed at high light intensity (Fig. 4). Finally, we predicted that within-leaf variation in stomatal size would correlate with stomatal spacing, as larger stomata can supply larger volumes of adjacent mesophyll. In all three light treatments, stomatal size positively co-varied with the stomatal zone, that is, adjacent region of mesophyll that would be supplied by that stomate (Fig. 6).

Stomatal spacing on *A. thaliana* leaves partially supports our overall hypothesis that natural selection minimizes the average distance between stomata and chloroplasts, for a given overall stomatal density. There are three non-mutually exclusive hypotheses for why several of our predictions were wrong. First, our predictions must be wrong because they are based on the overly simplistic assumption of a homogeneous porous medium within the mesophyll. Real leaf mesophylls are spatially heterogeneous and chloroplasts are distributed as discrete nodes. The intercellular air space conductance is determined by its porosity and tortuosity, both of which are heterogeneous within the leaf. The palisade is typically less porous than the spongy mesophyll (e.g. Théroux-Rancourt *et al.* 2017), which should impact the optimal patterning on stomata on ab- versus adaxial surfaces. Tortuosity is also systematically greater in the palisade in the lateral direction parallel to the leaf plane (Harwood *et al.* 2021). We might predict a greater coordination advantage of offset stomata by accounting for greater lateral tortuosity, but it is likely that benefit is still very small under realistic parameter space. Quantifying the patterns of heterogeneity in porosity, tortuosity and other factors (Earles *et al.* 2018) using 3D imaging (e.g. Borsuk *et al.* 2022) will be needed to generate more realistic hypotheses about optimal stomatal spacing.

Second, spatio-temporal variation of internal conditions within leaves and between stomatal responses may make uniform, coordinated stomatal surfaces less beneficial (Weyers and Lawson 1997; Lawson *et al.* 1998; Lawson and Weyers 1999). This is because our model assumes a uniform leaf, the internal conditions of which are periodic and solved empirically and, therefore, stable. Any horizontal concentration gradients due to environmental heterogeneity and variable induction times for interacting leaf processes may reduce the benefit of uniform stomatal patterning. Third, natural selection may be constrained by developmental processes that prevent phenotypes from reaching their adaptive optima. Stomatal development must be plastic to environmental cues interpreted through long-distance and cell-to-cell signalling pathways (Pillitteri and Torii 2012). This plasticity may come with the cost of being unable to orchestrate the development of an absolutely uniform stomatal grid. Fourth, the benefit of some traits may be of too little consequence to result in fitness differences large enough to respond to selection. We consider the plausibility of these alternative hypotheses below and present ideas for future work to test them.

We assume an idealized leaf epidermal and mesophyll structure that is homogeneous and unconstrained by other tradeoffs. Real leaves not only provide pathways for ${CO}_{2}$ diffusion but also must supply water, intercept light and deter herbivores and pathogens. All of these competing processes also happen on different time scales and can be observed as heterogeneity in stomatal density, aperture and internal leaf conditions across the leaf at any given moment (Lawson *et al.* 1998; Lawson and Weyers 1999). These competing interests result in heterogeneous epidermal and mesophyll structures that could alter predictions about optimal stomatal spacing. In order to maintain consistent leaf water potential across the lamina, stomatal density must be coordinated with vein density (Fiorin *et al.* 2016). Thus, stomatal spacing may be optimized not at the interstomatal level, but at a higher level, coordinating water transport and water loss. For example, the palisade mesophyll is more tightly packed than the spongy mesophyll as an adaptation to intercept light efficiently, so lateral diffusion may be more limiting in the adaxial portion of the leaf. This may explain why adaxial leaf surfaces have consistently higher dispersion indices than abaxial surfaces across all light treatments (Fig. 4). Future gas exchange models should incorporate heterogeneous mesophyll structures and hydraulic traits such as veins.

We are not aware of a developmental pathway that ensures an idealized placement of stomata on the leaf surface. Rather, stomatal development is a dynamic process that must be plastic to environmental cues. Leaves develop based on short- and long-distance signalling pathways that relay information about incoming light, humidity, temperature and surrounding stomata to developing leaf tissues (Pillitteri and Torii 2012). Our results show an intermediate level of dispersion in stomatal spacing may be best explained by these developmental pathways that ensure the proper spacing of stomata, with an added random effect brought about by the necessity for plasticity in stomatal development (Fig. 4). However, deviations from ideal stomatal spacing may be compensated for the simultaneous and coordinated development of the IAS (Baillie and Fleming 2020). The fact that stomata, which supply a greater mesophyll volume that tends to be larger, suggesting that plants may use coordinated development of multiple leaf anatomical features to compensate for nonideal stomatal spacing (Fig. 6).

In amphistomatous leaves, ideal stomatal spacing is complicated by a third dimension. Our gas exchange model demonstrates little photosynthetic gain from abaxial–adaxial stomatal coordination (Fig. 7). Even though lateral diffusion may limit photosynthesis (Morison *et al.* 2005), the marginal gain from optimally offsetting stomata is not sufficient to generate fitness differences relative to the strength of genetic drift (i.e. the drift-barrier). We can similarly extrapolate that an ideal, equilateral triangular stomatal spacing is only slightly better than a suboptimal pattern. Any benefit garnered by ideal stomatal spacing may be additionally offset by a cost to developmental flexibility in variable environments (Pillitteri and Torii 2012; Baillie and Fleming 2020). Explaining these observations as the result of weak selection is in tension with the finding that stomatal size and zone positively covary, which would suggest that small changes in lateral diffusion distance are significant. As described above, the positive correlation between stomatal size and zone may be explained by common developmental processes rather than as an adaptation to maximize ${CO}_{2}$ diffusion. In any case, there is no evidence for coordinated development of both leaf surfaces and very little theoretical benefit to photosynthesis, except in marginal circumstances that are exceptionally rare in nature.

Our study corroborates previous studies that demonstrate that stomata are non-randomly distributed along the leaf surface as a result of developmental mechanisms such as spatially biased arrest of stomatal initials (Boetsch *et al.* 1995), oriented asymmetric cell division (Geisler et al. 2000), and cell cycle controls (Croxdale 2000). We do not investigate the potential developmental pathways that influence stomatal dispersion in this study; however, they are important to consider as these pathways could limit plants from reaching a theoretical peak in the adaptive landscape: uniform stomatal patterning. Instead, as this study suggests, plants may simply compensate with higher stomatal density by modulating stomatal size to the area that they supply with ${CO}_{2}$. To understand why stomata are not ideally dispersed, more modelling (with more realistic assumptions including vein density and IAS structure) should be done to estimate the photosynthetic properties of varying stomatal patterning. Additionally, genetic manipulation studies should attempt to create mutants with clustered and uniformly patterned stomata for a comparison of their photosynthetic traits. This could have important implications for maximizing assimilation rates in crops as most crop species are grown in high light where ${CO}_{2}$ is often limiting. In drought-prone environments, increased stomatal dispersion may increase water use efficiency by reducing the number of stomata needed to achieve the same internal ${CO}_{2}$ concentration, $C_{\text{i}}$. However, it would be necessary to account for many other differences between *A. thaliana* and crop leaves and canopies.

Our results suggest that after optimizing stomatal density and having developmental rules for spacing stomata relatively evenly, there may be limited gains to further optimization. Therefore, developmental constraints may be necessary to make sense of some features of stomatal spacing and distribution. The possibility that ideal stomatal spacing is not the ‘tallest’ fitness peak must also be explored, as stomate size is demonstrated in this study to covary with mesophyll volume supplied with ${CO}_{2}$. This may be especially true in highly variable environments or in large tree species with sun and shade leaves where developmental cues may change rapidly. The temporal component, not considered here, could also have significant implications, as ${CO}_{2}$ may only be limiting to photosynthesis during short, relatively rare periods when all other conditions are ideal. In these cases, the theoretical benefits of ideal stomatal spacing are further diminished. Future exploration of these competing hypotheses would require more advanced modeling, additional exploration of IAS space development and its effects on gas exchange, both real and modelled, and knowledge about how often the species of interest is ${CO}_{2}$ limited across of range of natural settings. Despite these additional considerations, this study represents an important contribution to understanding the potential drivers of and limitations to stomatal anatomy in amphistomatous plants.

## Supplementary Material

plae015_suppl_Supplementary_Material

## Data Availability

Custom scripts are available on a GitHub repository (https://github.com/cdmuir/stomata-spacing) and archived on Zenodo: https://doi.org/10.5281/zenodo.10775962 Raw data are deposited on Dryad: https://doi.org/10.5061/dryad.44j0zpcn6

## Supporting Information

The following additional information is available in the online version of this article –

**Figure S1.** Example profiles of volumetric ${CO}_{2}$ concentrations within otherwise identical amphistomatous leaves that have stomatal positions offset (top row) or aligned (bottom row) based on the 2-D porous medium model.

**Table S1.** Glossary of model terms and mathematical symbols.
