# Supplementary material for: Does stomatal patterning in amphistomatous leaves minimize the CO2 diffusion path length within leaves?
Source: AoB Plants. 2024 Mar 20;16(2):plae015. doi: 10.1093/aobpla/plae015 (PMC11792893; doi:10.1093/aobpla/plae015)
Supplement: plae015_suppl_Supplementary_Material [file plae015_suppl_supplementary_material.pdf]

2-D Photosynthesis Model

We modeled leaf photosynthesis using a two-dimensional porous medium approximation. The `steady.2d()` function in the *R* package **rootSolve** version 1.8.2.4 (Soetaert and Herman 2009) solves the model using a finite element method (FEM). The 2-D leaf profile is n_x elements long and n_z elements deep with square elements of area t_{elem}^2. In all cases, we set $t_{\text{elem}} = 1$ μm. Table S1 is a glossary model terms and symbols.

Leaf anatomy

We assume that the leaf is a homogenous 2-D medium. The mesophyll is T_{leaf} thick and the stomata are regularly spaced apart by distance U on both ab- and adaxial surfaces. In this scenario, we assume that the stomata on each surface are precisely offset from each other by distance $U/2$. This minimizes the average distance between any point in the mesophyll and its nearest stomate. Because of the regular spacing, we only need to model the region between a stomate on surface and the next stomate on the other surface (Fig. S1). The rest of the mesophyll will be the same because of symmetry. This allowed us to set the boundary fluxes on the left and right sides of the leaf profile to 0.

Solving within-leaf gradients in CO_2 assimilation and concentration

We extended the 1-D FEM of Earles et al. (2017) to solve a set of partial differential equations describing CO_2 diffusion, photosynthesis, and respiration throughout a 2-D leaf geometry. The diffusive flux of CO_2 through ab- and adaxial stoamta, intercellular airspace, and mesophyll cells in the x (length) and z (depth) dimensions is:

$$D_{\text{e}} \nabla^2 C_{\text{ias}} = D_{\text{e}} \left(\frac{\partial^2 C_{\text{ias}}}{\partial x^2} + \frac{\partial^2 C_{\text{ias}}}{\partial z^2} \right) = -f_{\text{liq}}, \quad \text{(S1)}$$

where

$$f_{\text{liq}} = r_{\text{d}} + r_{\text{p}} - r_{\text{c}} \quad \text{(S2)}$$

and

$$D_e = \frac{\varphi}{\tau} D_c \tag{S3}$$

is the effective diffusivity of CO_2 though a porous medium composed of an intercellular airspace with a porosity (φ; m^3 airspace m^{-3} leaf) and tortuosity (τ; m m^{-1}). We assume the palisade (φ_{pal}) is less porous than the spongy (φ_{spg}) mesophyll (Table S1). D_c is the diffusion coefficient (m s^{-1}) for CO_2 in the intercellular airspace, C_{ias} is the [CO_2] (mol m^{-3}) at horizontal positions x and depth z in the intercellular airspace, f_{liq} is the volumetric rate of CO_2 diffusion from the intercellular airspace into the chloroplast stroma (mol m^{-3} s^{-1}), r_c is the volumetric rate of ribulose 1,5-bisphosphate (RuBP) carboxylation (mol m^{-3} s^{-1}), r_d is the volumetric respiration rate (mol m^{-3} s^{-1}), and r_p is the volumetric photorespiration rate by Rubisco (mol m^{-3} s^{-1}). Following Earles et al. (2017), r_d is assumed constant per stroma surface area (Table S1) and r_p is a function of carboxylation (r_c) and C_{liq}:

$$r_p = r_c \frac{\Gamma^*}{C_{liq}}. \tag{S4}$$

Carboxylation rate is the minimum of the Rubisco-limited (w_c) or RuBP-regeneration limited (w_j) carboxylation rates:

$$r_c = \min(w_c, w_j) \frac{S_m}{T_{leaf}} V_{strom}, \tag{S5}$$

where

$$w_c = \frac{k_c X_c C_{liq}}{K_m + C_{liq}}, \text{ and} \tag{S6}$$

$$w_j = \frac{C_{liq} j_e}{4C_{liq} + 8\Gamma^*}. \tag{S7}$$

Multiplying by $\frac{S_m}{T_{leaf}} V_{strom}$ converts carboxylation from per area to per stroma volume units. k_c is the catalytic rate of Rubisco (m^{-1}) and K_m is effective Michaelis-Menten constant for Rubisco (mol m^{-3}). Following Earles et al. (2017), we assumed the relative concentration of Rubisco follows that of Nishio, Sun, and Vogelmann (1993), but scaled such that the bulk leaf Rubisco concentration integrates to X_c described in Table S1. We estimated a continuous function describing the relative Rubisco profile as a function of leaf depth using a generalized additive model with the `gam()` function in *R* package **mgcv** version 1.9.0 (Wood 2017).

The effective photosynthetic e^- transport rate (j_e) is the minimum of the maximum (j_{max}) and potential (j_∞) photosynthetic e^- transport rates at each position within the mesophyll:

$$j_e = \min(j_{max}, j_\infty). \tag{S8}$$

The local j_{max} follows the same depth profile as Rubisco and is scaled by local S_m and V_{strom} so that it integrates to J_{max} on a leaf-area basis (Earles et al. 2017):

$$J_{max} = \int_0^{T_{leaf}} j_{max,i} S_{m,i} V_{strom} dz. \tag{S9}$$

Potential e$^-$ transport follows the same depth profile as chlorophyll concentration and is scaled by local S_m and V_{strom} so that it integrates to J_∞ on a leaf-area basis (Earles et al. 2017), where:

$$J_\infty = I_0 \alpha \beta \phi_{PSII} = \int_0^{T_{leaf}} j_{\infty,i} S_{m,i} V_{strom} dz. \tag{S10}$$

Potential e$^-$ transport is a product of PPFD incident on the leaf surface (I_0, mol m^{-2} s^{-1}), whole-leaf light absorption (α, mol mol^{-1}), the fraction of light absorbed by PSII (β, mol mol^{-1}), and the quantum yield of PSII e$^-$ transport (ϕ_{PSII}, mol mol^{-1}).

The local chlorophyll concentration is a function of depth described by a quadratic equation (Johnson et al. 2005):

$$F_{chl} = f(z) = b_{0,chl} + b_{1,chl} z + b_{2,chl} z^2. \tag{S11}$$

We used generic parameters from Borsuk and Brodersen (2019), rescaled to relative depth on a 0-1 rather than 0-100 interval.

For mass balance, the CO_2 assimilated via carboxylation must be supplied by diffusive flux from the intercellular airspace into the chloroplast stroma. The volumetric rate of CO_2 diffusion from the intercellular airspace into the chloroplast stroma, f_{liq}, is:

$$f_{liq} = \frac{g_{liq}(C_{liq} - C_{ias})}{T_{leaf}/S_m}, \tag{S12}$$

where g_{liq} is the CO_2 conductance from the intercellular airspace into the chloroplast stroma (m s^{-1}), C_{liq} (mol m^{-3}) is the [CO_2] in the stroma, and S_m is mesophyll surface area-to-leaf surface area ratio. Noting that g_{liq} is conductance per m^2 of stroma, this means the length scale to divide by should be the inverse of stroma area per unit bulk leaf volume, i.e. $1/[S_c(1/T_{leaf})] = T_{leaf}/S_c$. For simplicity, we assume that the entire mesophyll surface area is lined with chloroplasts, hence $S_m = S_c$. Following Earles et al. (2017), we assumed greater surface area in the palisade ($S_{m,pal}$) than spongy ($S_{m,spg}$) mesophyll. The fractions of palisade and spongy mesophyll are f_{pal} and f_{spg}, respectively.

Following Earles et al. (2017), we set boundary conditions at the stomata, C_{stom}, to be $0.85\times$ atmospheric CO_2 (Table S1). The fluxes on the left and right sides are 0 because of symmetry; the other fluxes on the ab- and adaxial surface are assumed 0 (i.e. the epidermis is impermeable to CO_2).

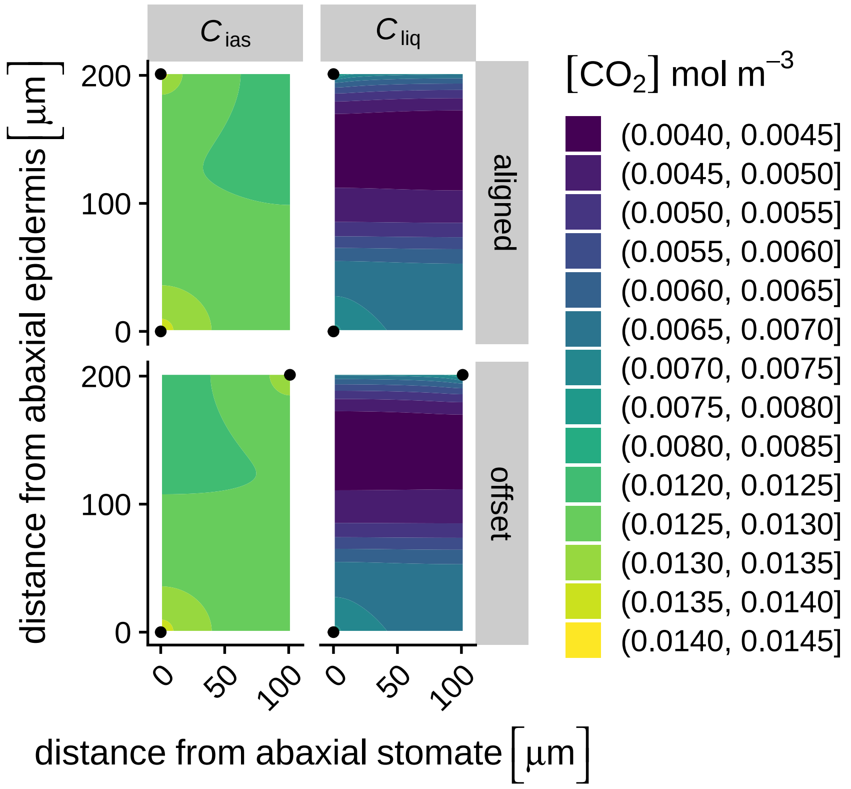

Figure S1: Example profiles of volumetric CO_2 concentrations within otherwise identical amphistomatous leaves that have stomatal positions offset (top row) or aligned (bottom row) based on the 2-D porous medium model. Stomatal positions are indicated by black points at the top and bottom of panels. When stomata are aligned, both ab- and adaxial stomata are position 0 along the x-axis; when stomata are offset, the adaxial stomate is positioned $U/2$ distance away. In this example, variables are set as: $I_0 = 1000$ μmol m^{-2} s^{-1}; $\varphi_{pal} = 0.2$ m^3 airspace m^{-3} leaf; $T_{leaf} = 200$ μm; $U = 200$ μm. All other parameter values are described in Table S1. C_{ias} = $[CO_2]$ in intercellular airspace; C_{liq} = $[CO_2]$ in chloroplast stroma; I_0 = PPFD incident on the leaf surface; φ_{pal} = Fraction of intercellular airspace (aka porosity), palisade; T_{leaf} = Leaf thickness; U = Interstomatal distance.

Table S1: Glossary of model terms and mathematical symbols.

Name	Symbol	Value	Units	Notes
Whole-leaf light absorption	α	0.8	mol mol^{-1}	assumed
Chlorophyll spatial distribution coefficient	$b_{0,\mathrm{chl}}$	67.5	—	Borsuk and Brodersen (2019); Equation S11
Chlorophyll spatial distribution coefficient	$b_{1,\mathrm{chl}}$	41.5	—	Borsuk and Brodersen (2019); Equation S11
Chlorophyll spatial distribution coefficient	$b_{2,\mathrm{chl}}$	−29	—	Borsuk and Brodersen (2019); Equation S11
Fraction of light absorbed by PSII	β	0.5	mol mol^{-1}	assumed
[CO_2] in intercellular airspace	C_{ias}	calculated	mol m^{-3}	Equation S1 and Equation S12
[CO_2] in chloroplast stroma	C_{liq}	calculated	mol m^{-3}	Equation S1
[CO_2] in substomatal cavity	C_{stom}	1.50×10^{-2}	mol m^{-3} leaf	assumed
[CO_2] compensation point	Γ^*	1.35×10^{-3}	mol m^{-3} stroma	Caemmerer (2000)
Diffusivity of [CO_2] in intercellular airspace	D_{c}	1.54×10^{-5}	m^2 s^{-1}	assumed
Effective diffusivity of [CO_2] in intercellular airspace	D_{e}	calculated	m^2 s^{-1}	Equation S3
Fraction of palisade mesophyll	f_{pal}	0.6	1	$1 = f_{\mathrm{pal}} + f_{\mathrm{spg}}$
Fraction of spongy mesophyll	f_{spg}	0.4	1	$1 = f_{\mathrm{pal}} + f_{\mathrm{spg}}$
Chlorophyll fluoresence profile along leaf depth normalized by total fluoresence	F_{chl}	calculated	1	Borsuk and Brodersen (2019); Equation S11
Conductance of cell wall, plasmalemma, cytosol, chloroplast envelope, and chloroplast stroma	g_{liq}	2.50×10^{-4}	m^3 m^{-2} stroma s^{-1}	Evans et al. (2009)
PPFD incident on the leaf surface	I_0	variable	mol m^{-2} s^{-1}	assumed
Potential photosynthetic e$^-$ transport rate on a leaf area basis	J_∞	calculated	mol m^{-2} leaf s^{-1}	Equation S10
Maximum photosynthetic e$^-$ transport rate on a leaf area basis	J_{max}	2.75×10^{-4}	mol m^{-2} leaf s^{-1}	assumed

Name	Symbol	Value	Units	Notes
Effective photosynthetic e^- transport rate on a stroma volume basis	j_e	calculated	mol m^{-3} stroma s^{-1}	Equation S8
Potential photosynthetic e^- transport rate on a stroma volume basis	j_∞	calculated	mol m^{-3} stroma s^{-1}	Equation S10
Maximum photosynthetic e^- transport rate on a stroma volume basis	j_{max}	calculated	mol m^{-3} stroma s^{-1}	Equation S9
Catalytic rate of Rubisco	k_c	2.84	m^{-1}	Tholen and Zhu (2011)
Rubisco effective K_m	K_m	1.87×10^{-2}	mol m^{-3}	Caemmerer (2000)
Number of elements in x direction	n_x	calculated	—	$U = 2n_x t_{elem}$
Number of elements in z direction	n_z	calculated	—	$T_{leaf} = n_z t_{elem}$
Fraction of intercellular airspace (aka porosity), palisade	φ_{pal}	variable	m^3 airspace m^{-3} leaf	assumed
Fraction of intercellular airspace (aka porosity), spongy	φ_{spg}	0.3	m^3 airspace m^{-3} leaf	assumed
Quantum yield of PSII e^- transport	ϕ_{PSII}	0.85	mol mol^{-1}	assumed
Volumetric rate of RuBP carboxylation	r_c	calculated	mol m^{-2} stroma s^{-1}	Equation S5
Volumetric respiration rate	r_d	6.60×10^{-2}	mol m^{-2} stroma s^{-1}	Earles et al. (2017); Tholen and Zhu (2011)
Volumetric rate of photorespiratory CO_2 release	r_p	calculated	mol m^{-2} stroma s^{-1}	Earles et al. (2017); Equation S4
Mesophyll surface area-to-leaf surface area ratio, palisiade	$S_{m,pal}$	20	m^2 mesophyll m^{-2} leaf	assumed
Mesophyll surface area-to-leaf surface area ratio, spongy	$S_{m,spg}$	2	m^2 mesophyll m^{-2} leaf	assumed
Tortuosity of intercellular airspace	τ	1.55	m m^{-1}	Syvertsen et al. (1995)
Thickness of element in both x and z directions	t_{elem}	1.00×10^{-6}	m	$T_{leaf} = n_z t_{elem}$

Name	Symbol	Value	Units	Notes
Leaf thickness	T_{leaf}	variable	m	$T_{\text{leaf}} = n_z t_{\text{elem}}$
Interstomatal distance	U	variable	m	$U = n_x t_{\text{elem}}$
Stroma volume-to-mesophyll surface area ratio	V_{strom}	1.74×10^{-6}	m^3 stroma m^{-2} mesophyll	Earles et al. (2017); Tholen and Zhu (2011)
Rubisco-limited carboxylation rate	w_c	calculated	mol m^{-2} stroma s^{-1}	Equation S6
RuBP regeneration-limited carboxylation rate	w_j	calculated	mol m^{-2} stroma s^{-1}	Equation S7
Rubisco concentration in stroma	X_c	2.5	mol m^{-3} stroma	Tholen and Zhu (2011); Oguchi, Hikosaka, and Hirose (2003)

References

Borsuk, Aleca M., and Craig R. Brodersen. 2019. "The Spatial Distribution of Chlorophyll in Leaves." *Plant Physiology* 180 (3): 1406–17. https://doi.org/10.1104/pp.19.00094.

Caemmerer, Susanne von. 2000. *Biochemical Models of Leaf Photosynthesis*. Techniques in Plant Science 2. Collingwood: CSIRO.

Earles, J. Mason, Guillaume Théroux-Rancourt, Matthew E. Gilbert, Andrew J. McElrone, and Craig R. Brodersen. 2017. "Excess Diffuse Light Absorption in Upper Mesophyll Limits CO_2 Drawdown and Depresses Photosynthesis." *Plant Physiology* 174 (2): 1082–96. https://doi.org/10.1104/pp.17.00223.

Evans, J. R., R. Kaldenhoff, B. Genty, and I. Terashima. 2009. "Resistances Along the CO_2 Diffusion Pathway Inside Leaves." *Journal of Experimental Botany* 60 (8): 2235–48. https://doi.org/10.1093/jxb/erp117.

Johnson, Daniel M., William K. Smith, Thomas C. Vogelmann, and Craig R. Brodersen. 2005. "Leaf Architecture and Direction of Incident Light Influence Mesophyll Fluorescence Profiles." *American Journal of Botany* 92 (9): 1425–31. https://doi.org/10.3732/ajb.92.9.1425.

Nishio, J. N., J. Sun, and T. C. Vogelmann. 1993. "Carbon Fixation Gradients Across Spinach Leaves Do Not Follow Internal Light Gradients." *The Plant Cell*, August, 953–61. https://doi.org/10.1105/tpc.5.8.953.

Oguchi, R., K. Hikosaka, and T. Hirose. 2003. "Does the Photosynthetic Light-Acclimation Need Change in Leaf Anatomy?: Chloroplast Volume Change in Photosynthetic Light-Acclimation." *Plant, Cell & Environment* 26 (4): 505–12. https://doi.org/10.1046/j.1365-3040.2003.00981.x.

Soetaert, Karline, and Peter M. J. Herman, eds. 2009. *A Practical Guide to Ecological Modelling*. Dordrecht: Springer Netherlands. https://doi.org/10.1007/978-1-4020-8624-3.

Syvertsen, J. P., J. Lloyd, C. McConchie, P. E. Kriedemann, and G. D. Farquhar. 1995. "On the Relationship Between Leaf Anatomy and CO_2 Diffusion Through the Mesophyll of Hypostomatous Leaves." *Plant, Cell and Environment* 18 (2): 149–57. https://doi.org/10.1111/j.1365-3040.1995.tb00348.x.

Tholen, D., and X.-G. Zhu. 2011. "The Mechanistic Basis of Internal Conductance: A Theoretical Analysis of Mesophyll Cell Photosynthesis and CO_2 Diffusion." *Plant Physiology* 156 (1): 90–105. https://doi.org/10.1104/pp.111.172346.

Wood, Simon N. 2017. *Generalized Additive Models: An Introduction with R*. 2nd ed. Chapman; Hall/CRC. https://doi.org/10.1201/9781315370279.
